# Endophytic Fungi from *Alstonia boonei* De Wild and *Greenwayodendron suaveolens* (Engl. and Diels) Verdc. subsp. *Suaveolens* Possess Inhibitory Activity against Pneumonia Causing Bacteria

**DOI:** 10.1155/2021/9966323

**Published:** 2021-08-05

**Authors:** Paola Cynthia Emoh Demeni, Patrick Hervé Diboue Betote, Christelle Wayoue Kom, Eric Ngalani Tchamgoue, Esther Del Florence Ndedi Moni, Jacqueline Saurelle Foumane Maniepi, Gabriel A. Agbor, Maximilienne Ascension Nyegue

**Affiliations:** ^1^Laboratory of Microbiology, Department of Microbiology, Faculty of Science, University of Yaoundé I, P.O. Box 812, Yaoundé, Cameroon; ^2^Laboratory of Pharmacology, Centre for Research on Medicinal Plants and Traditional Medicine, Institute of Medical Research and Medicinal Plants Studies, P.O. Box 13033, Yaoundé, Cameroon; ^3^Multidisciplinary Laboratory, Department of Galenical Pharmacy and Pharmaceutical Law, Faculty of Medicine and Biomedical Sciences, University of Yaoundé I, Yaoundé, Cameroon; ^4^Equipe Glyco et Nanovecteurs Pour le Ciblage Thérapeutique, Université of Montpellier, IBMM UMR 5247 CNRS-UM. 15 Avenue Charles Flahault, BP 14491, FR-34093 Montpellier Cedex 5, France

## Abstract

This study evaluated the antibacterial efficacy of methanolic extracts of isolated endophytic fungi from stem barks and leaves of *Alstonia boonei* De Wild and *Greenwayodendron suaveolens* (Engl. and Diels) Verdc. subsp. *Suaveolens* against *Klebsiella pneumoniae* ATCC 43816, *Haemophilus influenzae* ATCC 49247, *Pseudomonas aeruginosa* ATCC 27853, and *Escherichia coli* ATCC 35218, responsible for causing pneumonia. The endophytic fungi were isolated and characterized in the Potato Dextrose Agar (PDA), Sabouraud Dextrose Agar (SDA), and Czapek Dox Agar (CDA) media. The fungi and their methanolic extracts were tested for *in vitro* antibacterial potential by antagonistic assay for endophytic fungi against bacterial pathogens and microdilution method. The phytochemical screening of extracts was carried out according to the colorimetric and precipitation methods to reveal the presence of secondary metabolites. The results showed that 24 macroscopically and microscopically distinct endophytic fungi were isolated, identified, and stored. These endophytic fungi possessed antibacterial activity against the selected bacterial strains with inhibition zones ranging from 7.00 to 25.00 mm. The endophytic fungi GS_15_ and AB_24_ have presented the inhibitions zones of 20.33 mm and 25.00 mm, respectively, and these were better than the ones obtained for Levofloxacin®. The endophytes with inhibition zones greater than 10 mm were used for extraction of their secondary metabolites. The endophytic fungi extracts showed antibacterial activity with the minimum inhibitory concentrations (MICs) ranging from 6.25 × 10^−4^ to 2 × 10^−2^ g/L and the minimum bactericidal concentrations (MBCs) ranging from 2.5 × 10^−3^ to 2 × 10^−2^ g/L. The endophytic fungi GS_15_ extract was the most effective extract; it showed bactericidal effects on the tested bacterial strains. The phytochemical screening of the extracts revealed the presence of secondary metabolites classes, responsible for causing the obtained antibacterial activity. Thus, the endophytic fungi methanolic extracts from *A. boonei* and *G. suaveolens* have the potential to inhibit the growth of bacteria responsible for nosocomial pneumonia.

## 1. Introduction

Pneumonia is a lower respiratory tract infection caused by several infectious agents, including viruses, bacteria, and fungi [1]. However, pneumonia caused by bacterial infection has a rapidly progressive clinical course, which often becomes complicated by inflammation of the lungs, multilobular involvement, and lung abscesses [2, 3]. As the third deadliest infectious disease worldwide after tuberculosis and hepatitis B, it is also the leading cause of infectious deaths in children worldwide, but it is most prevalent in South Asia and sub-Saharan Africa. It accounted for 922,136 and 808,694 deaths in children below the age of five years in 2015 and 2017, respectively, representing up to 15% of all deaths of children below five years of age [4]. Hence, the pneumonia causing bacteria represents the leading cause of infant morbidity and mortality recorded in low-income countries [5] accounting for 16.5% deaths among children aged 0 to 59 months in Cameroon [6, 7]. In France, pneumonia is also responsible for 30% of deaths among adults over the age of 65 years. Appropriate antibiotic therapy involves Amoxicillin®/Clavulanic® acid and Levofloxacin® for patients with pneumonia [8]. However, this treatment is relatively expensive in low-income countries; meanwhile, the pneumonia causing Gram-negative bacteria is responsible for microbial resistance [9]. Therefore, this scenario necessitates exploration of the various possibilities for treatment of this disease [10]. Different therapeutic strategies have already been implemented for treatment of this disease; among which use of medicinal plants offers an inexhaustible source of drugs [11].

*Alstonia boonei* De Wild belongs to the Apocynaceae family. About 110 species of this genus grow alongside the American tropical region. Preparations using the leaves, seeds, stem barks, and roots of different plants from the Apocynaceae family have been largely used in traditional medicine and some plants of *Alstonia* genus have been widely used as a febrifuge to treat malaria and other skin problems. Alcoholic extracts of the stem barks of *A. boonei* (to a lesser degree of the leaf) showed a broad-spectrum activity against both Gram-negative and Gram-positive bacteria, as well as fungi [12].

According to Lissambou et al., *Greenwayodendron suaveolens* (Engl. and Diels) belonging to the Annonaceae family has long been used as food and herbal medicine in Central Africa, especially as powdered supplement [13]. The genus *Greenwayodendron* is traditionally used for its antimalarial, antimenorrhagic, and antidysenteric properties [14]. This genus is a rich source of biologically active secondary metabolites with antimicrobial [15, 16], analgesic [17], antimalarial [18], and anthelminthic [19] activities.

For centuries, plants have served as medicinal bioactive compounds source against many forms of disease. In contrast, during the last few years, microorganisms associated with plants rather than the plants themselves have been shown to offer products with high therapeutic potential [20]. All microorganisms (bacteria and fungi) that inhabit asymptomatically, at least for one period of their life cycle, the internal plant tissues beneath the epidermal cell layers may be considered as endophytes [21]. Their role is to improve plant's access to nutrients and produce special substances, mainly secondary metabolites and enzymes which are responsible for plant adaptation to abiotic stress [22]. During the long coevolution of endophytes and their host plants, endophytes have adapted themselves to their special microenvironments by genetic variation, including uptake of some plant DNA into their own genomes. This could have led to the ability of certain endophytes to biosynthesize some “phytochemicals” originally associated with the host plants [23]. Of the endophytic microorganisms, fungi have been isolated the most [24] as they have been proved to be the promising sources of biologically active products with antioxidant, immunosuppressive, antiproliferative, anti-inflammatory [23], and antibacterial activities [25]. Furthermore, considering the exponential evolution of antibiotic resistance in human pathogens in recent years, it has become inevitable to search for more efficacious antibiotics. Thus, the antimicrobial activities have been evaluated for a variety of metabolites biosynthesized by endophytic fungi [10, 24] and there is an increasing effort to characterize, identify, and evaluate the potential biological effects of endophytic fungal extracts isolated from medicinal plants [21]. However, the endophytes of *A. boonei* and *G. suaveolens* have not yet been characterized.

The present study evaluated the antibacterial potential of the methanolic extracts of endophytic fungi of stem barks and leaves of *Alstonia boonei* De Wild and stem barks of *Greenwayodendron suaveolens* (Engl. & Diels) Verdc. subsp. *Suaveolens* on the growth of several bacterial strains responsible for nosocomial pneumonia.

## 2. Material and Methods

### 2.1. Plant Materials

Healthy and mature plants without any visual disease symptom were carefully chosen for sampling. Leaves and stem barks of *A. boonei* were harvested from the botanical garden of the Institute of Medical Research and Medicinal Plants Studies, Yaoundé, Cameroon (Lat. 3° 51′ 39.298″ N; Long. 11° 30′ 19.192″ E) in the month of November 2018 and the stem barks of *G. suaveolens* were harvested in February 2019 at Mount Kala (Lat. 3° 52′ 28.515″ N; Long. 11° 27′ 9.335″ E) in Nkolbisson locality in Yaoundé, Cameroon. *A*. *boonei* and *G. suaveolens* samples were identified at the Cameroon National Herbarium under identification no. 43365/HNC and identification no. 45578/HNC, respectively.

### 2.2. Isolation and Cultivation of Endophytic Fungi

The plant samples were washed under running tap water, and sterilization of leaves surfaces was achieved by subsequently soaking them in a series of solutions as follows: sterile distilled water for 2 min, ethanol 70% for 2.5 min, sodium hypochlorite 2.4% for 4 min, and ethanol 70% for 1 min, and finally they were rinsed with sterile distilled water for 3 times [26, 27]. They were dried in sterile absorbent paper. The last washing water was plated on Petri dishes containing Potato Dextrose Agar (PDA). The success of surface sterilization method was confirmed by the absence of any microbial growth on the culture media plated with the last washing water. The sterilized leaves were cut into 5 mm segments using a sterilized knife. Ten parts of each plant segments were placed in Petri dishes (9 cm) containing PDA supplemented by 0.5% of chloramphenicol and incubated at 28 ± 2°C [26]. Regular observations were carried out from the second day onwards for a period of 3 to 4 weeks for fungal growth. The fungal growth from internal tissues was checked for purity and transferred to fresh culture slants and then stored at 4°C for further study [27].

### 2.3. Identification of Endophytic Fungi

Identification of endophytic fungi associated with leaves or stem barks segments of plants was done based on their morphological and taxonomic properties (macroscopically and microscopically) on PDA, Sabouraud Dextrose Agar (SDA), and Czapek Dox Agar (CDA) media at 28 ± 2°C [28–34]. The macroscopic characteristics observed were color and surface colonies (granular, such as flour, mounting, slippery), texture, zonation, growth area, the lines of radial and concentric, reverse color, and exudate drops. The microscopic examinations of the vegetative thallus, fruiting bodies, and spores were carried out with methylene blue and Congo red reagents. The species identification was done according to the methods described earlier [35–38].

### 2.4. Extraction of Secondary Metabolites of Endophytic Fungi

#### 2.4.1. Fermentation

Four endophytic fungi previously purified with inhibition zones higher than 10 mm on the tested bacteria were chosen for this purpose. Thus, some mycelial agar plugs from these endophytic fungi culture were cultivated into 500 mL culture flask containing 250 grams of sterile rice. The culture was incubated at still condition at room temperature under dark condition for 3 to 4 weeks [39].

#### 2.4.2. Extraction of Endophytic Fungal Culture

After incubation period, biomass and culture media were extracted with methanol. The methanolic fraction was evaporated under reduced pressure by Heidolph brand rotary evaporator.

### 2.5. Assessment of Antibacterial Activity

#### 2.5.1. Bacterial Strains

The antibacterial activities were tested using qualitative biological analysis in triplicate. The pathogenic bacteria used in this study were *Haemophilus influenza* ATCC 49247, *Pseudomonas aeruginosa* ATCC 27853, *Escherichia coli* ATCC 35218, and *Klebsiella pneumoniae* ATCC 43816. These bacterial strains were provided by the Laboratory of Bacteriology of Yaoundé University Hospital Centre (Cameroon).

#### 2.5.2. Antagonistic Assay for Endophytic Fungi against Bacterial Pathogens

Antagonistic activities of twenty-four endophytic fungi were tested for their antibacterial activity [40]. The test bacteria grown on liquid Mueller-Hinton medium for 24 h; the concentration was adjusted at 1 × 10^6^ cells/mL [41]. The bacteria (100 *μ*L) were inoculated on the Petri dishes containing solid Mueller-Hinton medium. Afterwards, the disks of agar (Ø 6 mm) of each endophytic fungus young strain (5 to 7 days) were inoculated and placed at equidistant. The plates remained incubated at 37 ± 2°C for 24 h. Levofloxacin® (Sigma) (100 *μ*g/mL in dimethyl sulfoxide (4%; v/v)) was employed as positive control. All the experiments were carried out using a completely randomized design (CRD), with three repetitions [42].

The presence of zone clearance on agar plates was used as an indicator for the antibacterial activity of endophytic fungi under investigation. The strains, which showed the maximum zone of clearance, were chosen for further study. The presence of these inhibition zones on agar plates was used as indicator of bioactive potential of endophytic fungi strains [43, 44].

#### 2.5.3. Microdilution Assay of Endophytic Fungi Extracts

*(1) Determination of Minimum Inhibitory Concentration (MIC)*. The geometric serial broth microdilution method was carried out according to the Microplate Alamar Blue Assay (MABA) described previously by the Clinical and Laboratory Standards Institute. A stock solution was prepared by diluting the respective sample in dimethyl sulfoxide (4%; v/v). Stock solution was then added to Mueller-Hinton broth to reach final samples concentrations ranging from 2 × 10^−2^ g/L to 1.95 × 10^−5^ g/L. Bacterial inocula (1.5 × 10^8^ CFU/mL) were added to each dilution. Each 96-well microtiter plate was incubated at 37°C for 24 h. Positive control consisted of Levofloxacin® at 2.5 × 10^−4^ g/L, negative control contained no drugs, and blank contained neither inoculum nor drug. The concentration of dimethyl sulfoxide in the assay was kept at 5% to ensure that its effect on bacterial growth can be maintained as minimal. Upon incubation periods, 40 *μ*L of 0.02% resazurin was added to individual wells and the plates were reincubated for additional 30 min and checked for color change. Change in resazurin color from blue to pink indicated reduction of the indicator due to bacterial growth. The MIC was defined as the lowest concentration of samples at which the microorganisms did not demonstrate growth [45].

*(2) Determination of Bactericidal Effect*. The Minimal Bactericidal Concentrations (MBC) of promising methanolic extracts of endophytic fungi were assessed by subculturing MIC test microtiter plates on Mueller-Hinton medium. The MBC was considered as highest dilution or lowest concentration at which no growth occurred in the medium. All the experiments were done in triplicate [45].

The antibacterial effect was deemed bactericidal or bacteriostatic depending on the ratio of MBC to MIC. If MBC/MIC is lower than four, the antibacterial effect of endophytic fungi extracts is bactericidal and bacteriostatic when MBC/MIC was higher than four [46].

### 2.6. Phytochemical Screening of Endophytic Fungi Extracts

Preliminary phytochemical screening of secondary metabolites of methanolic extracts of endophytic fungi was carried out according to methods described by Harborne and Evans [47, 48].

### 2.7. Statistical Analysis

All experiments for antagonistic assays were conducted in triplicate and values of inhibition zones expressed in mm as the mean ± SD. Variations in means were analyzed using one-way analysis of variance (ANOVA) and means were statistically significant if *p* < 0.05.

## 3. Results

### 3.1. Isolation and Identification of Endophytic Fungi

A total of 83 endophytic fungi were isolated from *A. boonei* and *G*. *suaveolens* and grouped into 24 fungi isolates. It was found that 16 fungi isolates were identified, belonging to four different genera: *Aspergillus* (12.5%), *Fusarium* (37.5%), *Neoscytalidium* (8.33%), and *Acremonium* (8.33%). These genera belong to Deuteromycota division, more precisely to the Hyphomycetes class. They were divided into hyaline hyphomycetes (Moniliaceae order) with *Aspergillus* spp., *Fusarium* spp., *Acremonium* spp., and dark hyphomycetes (Dematiaceae order) with the only genus *Neoscytalidium* spp. being identified. However, the remaining 08 endophytic fungi could not be identified due to their issues related infertility (Table 1).

**Figure 1:**
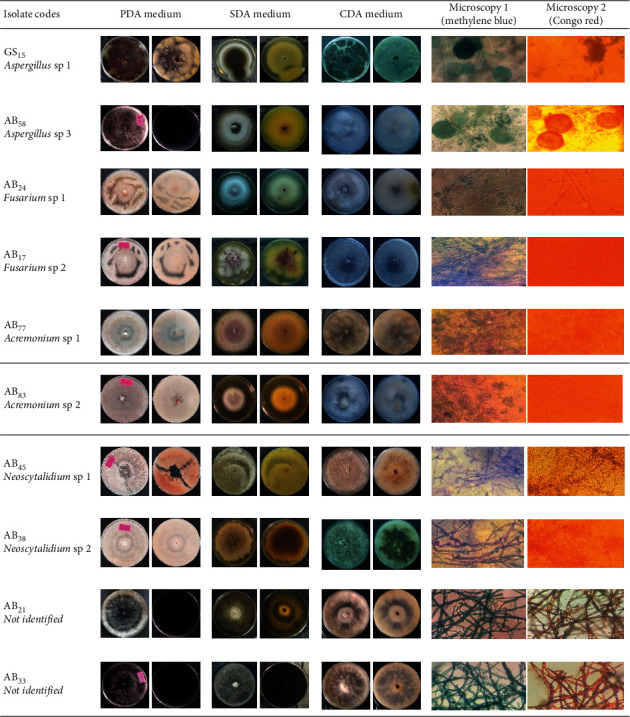
Morphology and microscopic characters of endophytic fungi isolated from *Alstonia boonei* and *Greenwayodendron suaveolens*.

Some morphological and microscopical characteristics of isolated endophytic fungi are represented in Figure 1.

Isolates of the genus *Aspergillus* appeared with bright colors, which made their identification easy. Three species of this genus were identified in this study. The mycelial colonies of the isolated species are either powdery with black color (*Aspergillus* sp. 1) and green in PDA and SDA media but orange-yellow in CDA medium (*Aspergillus* sp. 3) or downy with black color in PDA and white in CDA and SDA media (*Aspergillus* sp. 2). These isolates were also characterized by a septate thallus and an unbranched conidiophore with varying lengths and shapes, ending in a bulge or vesicle bearing phialides or sometimes separated by metules, which is characteristic of the genus called *Aspergillus* head.

The colonies of isolates of the *Fusarium* genus were downy or cottony with different colors ranging from pinkish white for *Fusarium* sp. 1 and 2 to brown in PDA for *Fusarium* sp. 3 with other colors such as pink or yellow for *Fusarium* spp. 4, 7, 8, and 9, especially in SDA and CDA media. Microscopy presented septate vegetative thallus with cluster or chain arrangement of microconidia, fusiform macroconidia, curved and quite pointed at the tips of the phialides, and the early presence of chlamydospores as seen on *Fusarium* sp. 2, justifying their belonging to the *Fusarium* genus. The colonies of isolates of the *Acremonium* genus were woolly in SDA and CDA and very poor in filaments or even hairless in PDA. Different colors were obtained like white and pink in SDA medium with a slow growth rate for *Acremonium* sp. 2 and a rapid growth rate for *Acremonium* sp. 1, as well as milky-white and white in PDA and CDA media. Microscopy presented a vegetative thallus made up of septate filaments, fine, cylindrical phialides at their tips, and cylindrical conidia grouped together in clusters. The colonies produced by the genus *Neoscytalidium* were milky-white fluffy in PDA and CDA media or woolly beige for *Neoscytalidium* sp. 2 and hairless brown for *Neoscytalidium* sp. 1 in SDA media. Microscopically, the hyphae are septate and hyaline; others are larger and strongly pigmented dissociating into arthroconidia. However, fungi isolates such as 17, 18, and 19 were unidentified as they did not produce sporing structures on PDA, SDA, and CDA media but presented dirty white cotton growth that darkened with age differently in each isolate. Microscopy presented septate and dark hyphae in fungi 17 and 18 differently from fungus 19, which presented septate and hyaline hyphae.

### 3.2. Antagonistic Potential of Endophytic Fungi

In this investigation, four endophytic fungi (GS_15_, AB_24_, AB_38_, and AB_83_) were selected for determination of inhibition parameters (MIC and MBC) after testing the 24 fungi isolates from *A. boonei* and *G. suaveolens* on the growth of *H. influenza*, *P. aeruginosa*, *E. coli*, and *K. pneumoniae*. These fungi isolates (GS_15_, AB_24_, AB_38_, and AB_83_) presented an antibacterial activity, with the inhibition zones ranging from 10.33 ± 0.57 mm against *E. coli* to 25.00 ± 1.00 mm against *H. influenza*. This was the most sensitive bacteria compared to those selected endophytic fungi with inhibition zone ranging from 20.33 ± 0.57 mm for AB_24_ to 25.00 ± 1.00 mm for GS_15_; the data obtained was compared to Levofloxacin® with an inhibition zone of 19.05 ± 0.82 mm as in Table 2.

### 3.3. Antibacterial Activity of Endophytic Fungi Extracts

The antibacterial activities of the methanolic extracts of endophytic fungi from *A. boonei* and *G. suaveolens* are presented in Table 3. The methanolic extracts presented antibacterial activity against *H. influenza*, *P. aeruginosa*, *E. coli*, and *K. pneumoniae* with MIC values of 6.25 × 10^−4^–2 × 10^−2^ g/L. The GS_15_ methanolic extract of endophytic fungi from *G. suaveolens* had the highest antibacterial activity with the MIC values of 6.25 × 10^−4^ g/L (*H. influenzae*), 2.5 × 10^−3^ g/L (*P. aeruginosa*), 2.5 × 10^−3^ g/L (*K. pneumoniae*), and 5 × 10^−3^ g/L (*E. coli*). The GS_15_ methanolic extract is the only extract that presented a bactericidal effect on the growth of the four bacteria strains studied. AB_38_ also presented a bactericidal effect on *H. influenzae* compared to the bacteriostatic effect obtained for Levofloxacin® on the same bacteria.

### 3.4. Phytochemical Screening

The extracts of the selected endophytic fungi (GS_15_, AB_24_, AB_38_, and AB_83_) have been used for several phytochemical tests to determine the presence of different types of bioactive compounds in them. The results presented in Table 4 indicated the presence of 10 secondary metabolites families in the methanolic extracts: alkaloids, phenols, polyphenols, tannins, saponins, flavonoids, anthocyanins, coumarins, terpenoids, and sterols. Unlike other classes of secondary metabolites that were ubiquitous to all four methanolic extracts, tannins were found exclusively in AB_38_ methanolic extract of *Neoscytalidium* sp. 2.

## 4. Discussion

### 4.1. Morphological and Microscopical Characterization

Isolation and identification of endophytic fungi have highlighted 4 genera, *G. suaveolens* (*Aspergillus*) and *A. boonei* (*Aspergillus*, *Acremonium*, *Neoscytalidium*, and *Fusarium*). The obtained results are similar to those of Abdel-Motaal et al. [49] who isolated endophytic fungi of *Neoscytalidium*, *Aspergillus*, *Acremonium*, and *Fusarium* genera from *Hyoscyamus muticus*, an Egyptian plant belonging to the Apocynaceae family same as *A. boonei*. In contrast, the study of Tolulope et al. [50] reported that the isolation of *A. boonei* endophytic fungi harvested in Nigeria contained three characterizing genera (*Aspergillus*, *Microphoma*, and *Trichoderma*). These results could therefore justify the hypothesis that endophytic fungi isolated from plants belonging to the same genus or family belong to the same genera. This is the case of *A. scholaris* and *Hyoscyamus muticus* according to the studies of Mahapatra and Banerjee and Abdel-Motaal et al., respectively, which indicated the presence of 19 genera with four similar genera (*Neoscytalidium*, *Aspergillus*, *Acremonium*, and *Fusarium*) in agreement with our study [49, 51]. We noticed that the colonizing endophytic fungi from *A. boonei* and *G. suaveolens* in our study have low genera diversities.

In addition, the genus *Fusarium* was the predominant genus isolated from *A. boonei* and *G. suaveolens* with a frequency of 37.5%. Indeed, it is one of the genera most associated with higher plants. *Fusarium* is among the most common fungi in terrestrial ecosystems. They are found in cultivated soils in temperate and tropical regions [37], and their predominance among the fungi isolated in this study corroborates with the study of Ilyas et al. [52] who explained that several fungi of the genus *Fusarium* were plant colonizers. In addition, *A. boonei* was harvested in tropical areas, where soils are favorable for the growth of species of this genus.

### 4.2. Antibacterial Activity of Endophytic Fungi Extracts

#### 4.2.1. Antagonistic Potential of Endophytic Fungi

The antagonistic potential of endophytic fungi (GS_15_, AB_24_, AB_38_, and AB_83_) allowed obtaining clearance zones on agar plates against *H. influenzae*, *P. aeruginosa*, *K. pneumoniae*, and *E. coli* which are greater than 10.33 mm on the bacterial strains tested. The present inhibition justifies the antibacterial activity of these endophytic fungi strains on the bacteria responsible for pneumonia, which could be due to the presence of bioactive substances in the mycelial discs of these endophytic fungi [53]. However, other studies have shown that endophytic fungi isolated from plants could have antibacterial activity, so they would resist invasion and inhibit the bacteria inducing the diseases in humans through the production of inhibitory substances belonging to several structural classes like alkaloids, peptides, steroids, terpenoids, phenols, quinines, and flavonoids [24, 54, 55]. Our results corroborate with an earlier report in which the endophytic fungi studied belonged to *Aspergillus*, *Acremonium*, and *Trichosporon* genera isolated from *Artemisia absinthium*. These endophytic isolates showed antibacterial activity against *P. aeruginosa* ATCC27853 and *E. coli* ATCC25922 strains [56]. Although the tested strains responsible for pneumonia are naturally resistant due to their “Gram-negative” character, the obtained results show that the bioactive compounds secreted by these endophytic fungi would have the capacity to penetrate the external membranes surrounding the wall of Gram-negative bacteria made up of covering lipopolysaccharides by the phenomena of diffusion of lipophilic compounds. Another phenomenon, which could also explain the results obtained, is the diffusion of active substances on the agar, which depends on the nature of the diffusing substances excreted by the endophytic fungi, their concentrations, their solubility, and pH of the culture medium [57]. The high antibacterial activity revealed by endophytic fungi isolated from *A. boonei* and *G. suaveolens* is related to their ability to produce bioactive substances with medicinal properties [58].

#### 4.2.2. Antibacterial Activity of Endophytic Fungi Extracts

The determination of the inhibition parameters (MIC and MBC) of methanolic extracts is shown by their MICs values of 6.25 × 10^−4^ to 2 × 10^−2^ g/L for *H. influenzae*, *P. aeruginosa*, *E. coli*, and *K. pneumonia* strains. The highest antibacterial activity was obtained with the methanolic extract of *Aspergillus* sp. 1 that presented a bactericidal effect on all tested strains. This could be linked to a synergistic or additive effect of the secondary metabolites present in the methanolic extract. In addition, the mean bactericidal effect of this extract on the strains tested is in line with data reported by Amadi and Adeniyi, as well as those reported by Indira et al. [59, 60] who demonstrated the antimicrobial activity of the genus *Aspergillus*. The reduction mechanisms of bacteria or fungi growth of the *Aspergillus* genus (*Aspergillus terreus*, *Aspergillus flavus*, and *Aspergillus fumigatus*) endophytes may possess higher concentration of alkaloids (fumiclavin A and B and, fumigaclavin B). In addition, most of these complex structures of fungi metabolites inhibit cell division and glucose transport [59, 61]. The data obtained in the present study were different from those of Kalhouche and Meziane [62] showing the MIC value of *Aspergillus* sp. 1 extract against *E. coli* to be 100 mg/mL. This difference may be because the *Aspergillus* strain used to inhibit the *E. coli* growth was not isolated from a medicinal plant. Hence, the production of secondary metabolites may not have been directed to any phytoprotective activity of plants. The methanolic extracts of AB_24_, AB_38_, and AB_83_ exhibited MICs ranging from 10^−2^ to 2 × 10^−2^ g/L. Indeed, the phytochemical screening of these extracts revealed the presence of phenols, flavonoids, and coumarins whose antibacterial effect could have been inhibited or neutralized by other compounds present in these extracts at higher concentrations [63]. These bioactive secondary metabolites would have the ability to penetrate the lipopolysaccharides of the wall of the Gram-negative bacteria tested and inhibit their growth by mechanisms of action that allowed them to inactivate bacterial enzymes, modify the structure of the active site, and induce intracellular accumulation of bioactive metabolites [64]. The bioactive compounds found in the methanolic extracts of endophytic fungi of *A. boonei* and *G. suaveolens* are responsible for the antibacterial activity and may have a synergistic action [65].

### 4.3. Phytochemical Screening of Endophytic Fungi Extracts

The phytochemical screening of the methanolic extracts of endophytic fungi isolated from *A. boonei* and *G. suaveolens* has shown that all the extracts contain alkaloids, polyphenols, flavonoids, terpenes, and sterols. The presence of these secondary metabolites in the endophytic fungi could justify their antibacterial effect. Previous studies have shown that phytochemical compositions of *A. boonei* and *G. suaveolens* stem barks extracts were similar to those in the present study with the presence of alkaloids, phenols, polyphenols, flavonoids, anthocyanins, saponins, terpenoids, and sterols [66, 67].

These studies permitted highlighting the phytomolecular mimicry of endophytic fungi extract from *A. boonei* and *G. suaveolens*. It appears that the phytochemical compositions of the endophytic fungi methanolic extracts of these plants would be similar to those of plant extracts [66, 67]. Therefore, to verify this hypothesis, it would be necessary in a further study to make additional quantitative and qualitative analysis, especially molecular and genetic levels between endophytic fungi and the host plant.

## 5. Conclusion

In this study, the endophytic fungi from *A. boonei* and *G. suaveolens* exhibited antibacterial activity against some of the human pathogenic bacteria responsible for nosocomial pneumonia. This activity was greater in the methanolic extract of GS_15_ endophytic fungal isolated from *G. suaveolens*, a plant that has never been listed for its antipneumonic potential. The present study helped to valorize the use of endophytic fungi from *A. boonei* and *G. suaveolens* in drug discovery process. However, a molecular identification of isolated endophytic fungi from these plants is necessary for further investigations.

## Figures and Tables

**Table 1:** Summary of endophytic fungi isolated from *Alstonia boonei* and *Greenwayodendron suaveolens*.

No.	Codes	Sources	Host plant	Genera names
01	GS_15_	Stem bark	*G. suaveolens*	*Aspergillus* sp. 1
02	GS_81_	Stem bark	*G. suaveolens*	*Aspergillus* sp. 2
03	AB_58_	Stem bark	*A. boonei*	*Aspergillus* sp. 3
04	ab_24_	Stem bark	*A. boonei*	*Fusarium* sp. 1
05	ab_17_	Stem bark	*A. boonei*	*Fusarium* sp. 2
06	ab_34_	Stem bark	*A. boonei*	*Fusarium* sp. 3
07	ab_46_	Stem bark	*A. boonei*	*Fusarium* sp. 4
08	ab_50_	Leave	*A. boonei*	*Fusarium* sp. 5
09	ab_59_	Stem bark	*A. boonei*	*Fusarium* sp. 6
10	ab_57_	Stem bark	*A. boonei*	*Fusarium* sp. 7
11	ab_80_	Stem bark	*A. boonei*	*Fusarium* sp. 8
12	ab_47_	Leave	*A. boonei*	*Fusarium* sp. 9
13	ab_77_	Stem bark	*A. boonei*	*Acremonium* sp. 1
14	ab_83_	Leave	*A. boonei*	*Acremonium* sp. 2
15	ab_45_	Leave	*A. boonei*	*Neoscytalidium* sp. 1
16	ab_38_	Stem bark	*A. boonei*	*Neoscytalidium* sp. 2
17	ab_21_	Stem bark	*A. boonei*	*Not identified* 1
18	ab_33_	Stem bark	*A. boonei*	*Not identified* 2
19	ab_36_	Leave	*A. boonei*	*Not identified* 3
20	ab_65_	Leave	*A. boonei*	*Not identified* 4
21	ab_23_	Stem bark	*A. boonei*	*Not identified* 5
22	ab_69_	Stem bark	*A. boonei*	*Not identified* 6
23	ab_78_	Stem bark	*A. boonei*	*Not identified* 7
24	ab_72_	Leave	*A. boonei*	*Not identified* 8

AB: *Alstonia boonei*; GS: *Greenwayodendron suaveolens*.

**Table 2:** Inhibition zones produced by endophytic fungi isolates on the growth of *K. pneumoniae*, *H. influenzae*, *P. aeruginosa*, and *E. coli*.

Endophytic fungi isolates	Inhibition zones (mm)			
	*H. influenzae*	*K. pneumoniae*	*P. aeruginosa*	*E. coli*
GS_15_	20.33 ± 1.52	19.00 ± 0.00	15.33 ± 0.57	10.33 ± 0.57
AB_17_	22.66 ± 0.57	22.66 ± 0.57	7.00 ± 0.00	7.00 ± 0.00
AB_21_	21.00 ± 1.00	17.66 ± 0.57	—	—
AB_23_	24.66 ± 0.57	19.00 ± 0.00	—	8.33 ± 0.57
AB_24_	25.00 ± 1.00	23.00 ± 0.00	20.00 ± 0.00	19.00 ± 0.00
AB_33_	—	—	—	—
AB_34_	17.33 ± 0.57	18.00 ± 0.00	8.33 ± 1.52	8.00 ± 0.00
AB_36_	10.66 ± 0.57	—	7.00 ± 0.00	—
AB_38_	20.33 ± 0.57	18.66 ± 0.57	19.00 ± 0.00	13.00 ± 1.00
AB_45_	14.00 ± 1.00	15.33 ± 1.52	—	17.33 ± 0.57
AB_46_	—	14.66 ± 1.52	8.00 ± 0.00	19.33 ± 0.57
AB_47_	7.00 ± 0.00	15.33 ± 1.15	—	18.33 ± 1.15
AB_50_	—	—	18.66 ± 1.52	—
AB_57_	13.00 ± 1.00	—	11.00 ± 1.00	16.00 ± 0.00
AB_58_	9.33 ± 0.57	—	—	—
AB_59_	—	10.66 ± 1.15	—	18.66 ± 0.57
AB_65_	—	12.00 ± 1.00	9.00 ± 0.00	17.00 ± 1.00
AB_69_	—	9.66 ± 0.57	—	8.00 ± 1.00
AB_72_	21.33 ± 1.15	—	—	7.00 ± 1.00
AB_77_	18.00 ± 1.00	—	8.00 ± 1.00	10.33 ± 0.57
AB_78_	8.33 ± 0.57	18.00 ± 0.57	7.00 ± 1.00	9.00 ± 1.00
AB_80_	24.00 ± 0.00	21.00 ± 1.00	9.00 ± 1.00	7.00 ± 0.00
GS_81_	16.00 ± 0.00	—	18.00 ± 1.00	13.00 ± 0.00
AB_83_	23.33 ± 0.57	19.66 ± 1.15	15.00 ± 1.00	12.66 ± 0.00
Levofloxacin®	19.05 ± 0.82	19.51 ± 0.35	27.57 ± 6.74	20.37 ± 1.64

AB: *Alstonia boonei*; GS: *Greenwayodendron suaveolens*; —: not determined. Values are the means of three replicates ± SD.

**Table 3:** Minimum inhibitory concentration (MIC) and minimum bactericidal concentration (MBC) for antibacterial activity of methanolic extracts of endophytic fungi from *A. boonei* and *G. suaveolens* against *K. pneumoniae*, *H. influenzae*, *P. aeruginosa*, and *E. coli*.

Samples	Bacterial strains	Inhibition parameters (g/L)			Antibacterial effects
		MIC	MBC	MBC/MIC	
GS_15_	*H. influenzae*	6.25 × 10^−4^	2.5 × 10^−3^	4	Bactericidal
	*P. aeruginosa*	2.5 × 10^−3^	5 × 10^−3^	2	Bactericidal
	*K. pneumoniae*	2.5 × 10^−3^	5 × 10^−3^	2	Bactericidal
	*E. coli*	5 × 10^−3^	2 × 10^−2^	4	Bactericidal

AB_24_	*H. influenzae*	2 × 10^−2^	>2 × 10^−2^	—	—
	*P. aeruginosa*	2 × 10^−2^	>2 × 10^−2^	—	—
	*K. pneumoniae*	2 × 10^−2^	>2 × 10^−2^	—	—
	*E. coli*	>2 × 10^−2^	—	—	—

AB_38_	*H. influenzae*	10^−2^	2 × 10^−2^	2	Bactericidal
	*P. aeruginosa*	10^−2^	>2 × 10^−2^	—	—
	*K. pneumoniae*	10^−2^	>2 × 10^−2^	—	—
	*E. coli*	2 × 10^−2^	>2 × 10^−2^	—	—

AB_83_	*H. influenzae*	2 × 10^−2^	>2 × 10^−2^	—	—
	*P. aeruginosa*	2 × 10^−2^	>2 × 10^−2^	—	—
	*K. pneumoniae*	2 × 10^−2^	>2 × 10^−2^	—	—
	*E. coli*	2 × 10^−2^	>2 × 10^−2^	—	—

Levofloxacin®	*H. influenzae*	78 × 10^−7^	625 × 10^−7^	8	Bacteriostatic
	*P. aeruginosa*	25 × 10^−5^	25 × 10^−5^	1	Bactericidal
	*K. pneumoniae*	>25 × 10^−5^	—	—	—
	*E. coli*	>25 × 10^−5^	—	—	—

AB: *Alstonia boonei*; GS: *Greenwayodendron suaveolens*; GS_15_: methanolic extract of *Aspergillus* spp. 1; AB_24_: methanolic extract of *Fusarium* spp. 1; AB_38_: methanolic extract of *Neoscytalidium* spp. 2 and AB_83_: methanolic extract of *Acremonium* spp. 2; —: not determined.

**Table 4:** Phytochemical screening of the methanolic extracts of endophytic fungi (GS_15_, AB_24_, AB_38_, and AB_83_).

Secondary metabolites	Endophytic fungi extracts			
	GS_15_	AB_24_	AB_38_	AB_83_
Alkaloids	+++	++	+	+
Phenols	−	+++	++	++
Polyphenols	++	+	+	+
Flavonoids	+	++	++	++
Tannins	−	−	+	−
Anthocyanins	−	+	+	+
Coumarins	++	+	−	++
Saponins	−	+	++	++
Terpenoids	+++	+	+	+
Steroids	+++	+	+	+

AB: *Alstonia boonei*; GS: *Greenwayodendron suaveolens*; GS_15_: methanolic extract of *Aspergillus* sp. 1; AB_24_: methanolic extract of *Fusarium* sp. 1; AB_38_: methanolic extract of *Neoscytalidium* sp. 2 and AB_83_: methanolic extract of *Acremonium* sp. 2.

## Data Availability

The datasets used and/or analyzed during the current study are available from the corresponding author upon reasonable request.
